# RSAtrace3D: robust vectorization software for measuring monocot root system architecture

**DOI:** 10.1186/s12870-021-03161-9

**Published:** 2021-08-25

**Authors:** Shota Teramoto, Takanari Tanabata, Yusaku Uga

**Affiliations:** 1 Institute of Crop Science, National Agriculture and Food Research Organization, 2-1-2, Kannondai, Tsukuba, Ibaraki, 305-8518 Japan; 2 Kazusa DNA Research Institute, 2-6-7, Kazusa-Kamatari, Kisarazu, Chiba, 292-0818 Japan

**Keywords:** Image analysis, 3D volume, Three-dimensional analysis, Python, Root length, Root growth angle, Root distribution

## Abstract

**Background:**

The root distribution in the soil is one of the elements that comprise the root system architecture (RSA). In monocots, RSA comprises radicle and crown roots, each of which can be basically represented by a single curve with lateral root branches or approximated using a polyline. Moreover, RSA vectorization (polyline conversion) is useful for RSA phenotyping. However, a robust software that can enable RSA vectorization while using noisy three-dimensional (3D) volumes is unavailable.

**Results:**

We developed RSAtrace3D, which is a robust 3D RSA vectorization software for monocot RSA phenotyping. It manages the single root (radicle or crown root) as a polyline (a vector), and the set of the polylines represents the entire RSA. RSAtrace3D vectorizes root segments between the two ends of a single root. By utilizing several base points on the root, RSAtrace3D suits noisy images if it is difficult to vectorize it using only two end nodes of the root. Additionally, by employing a simple tracking algorithm that uses the center of gravity (COG) of the root voxels to determine the tracking direction, RSAtrace3D efficiently vectorizes the roots. Thus, RSAtrace3D represents the single root shape more precisely than straight lines or spline curves. As a case study, rice (*Oryza sativa*) RSA was vectorized from X-ray computed tomography (CT) images, and RSA traits were calculated. In addition, varietal differences in RSA traits were observed. The vector data were 32,000 times more compact than raw X-ray CT images. Therefore, this makes it easier to share data and perform re-analyses. For example, using data from previously conducted studies. For monocot plants, the vectorization and phenotyping algorithm are extendable and suitable for numerous applications.

**Conclusions:**

RSAtrace3D is an RSA vectorization software for 3D RSA phenotyping for monocots. Owing to the high expandability of the RSA vectorization and phenotyping algorithm, RSAtrace3D can be applied not only to rice in X-ray CT images but also to other monocots in various 3D images. Since this software is written in Python language, it can be easily modified and will be extensively applied by researchers in this field.

## Background

As one of the elements that comprises root system architecture (RSA) [1, 2], root distribution in the soil determines uptake availability of water and nutrients in the soil. This is because plants could take up water and nutrients within the reach of their roots [3, 4]. Thus, root distribution greatly affects crop productivity [5]. For example, shallow-rooted plants perform better by utilizing fertilization close to the ground surface, whereas deep-rooted plants perform better by utilizing fertilization in the deep soil area [6]. In deep-rooted plants, water defects caused by drought are avoidable because their roots elongate into deeper soil zone containing more water [4, 7, 8].

Since RSA phenotyping processes are complex and laborious [9], RSA traits are not so widely measured compared to above-ground traits that are obviously related to the yield [10]. Another issue of RSA phenotyping is that most previously conducted RSA studies applied two-dimensional (2D) information to date [10, 11]. The 2D information would be insufficient to represent the three-dimensional (3D) root structure and deployment in the soil. Recently, 3D RSA phenotyping has been available using 3D imaging machines, such as X-ray computed tomography (CT), magnetic resonance imaging (MRI), and multicamera systems [11–14]. Particularly, X-ray CT and MRI could observe the roots in the soil in situ, and they have been extensively utilized for conducting RSA studies [15].

By utilizing software to quantify RSA traits, root segments in the X-ray CT and MRI images are isolated. In the case of X-ray CT, RooTrak [16, 17], RootViz3D® [18], Rootine [19], and RSAvis3D [20] were developed as software to segment roots in the soil. Based on the structure information such as root shape and branching, RooTrak and RootViz3D® isolate root segments by tracking the root segments from the point where an operator indicated [16–18]. In addition, Rootine and RSAvis3D isolate root segments without manual operation; Rootine segments roots using feature detection of the tubular shape of roots, while RSAvis3D segments roots using an edge detection algorithm [19, 20]. In the case of MRI, NMRooting software was developed [21]. NMRooting simply isolates root segments by a thresholding method [21]. Furthermore, RSA traits are measured based on the isolated root segments. For example, the root volume is calculated by the voxel counting of the root segments [16–18], while the root length is approximated by the voxel counting of the skeletonized root segments [19, 22].

RSA vectorization (connecting the adjacent root voxels to enable path to represent RSA shape) is a method of RSA phenotyping, allowing for topological analysis and root-growing angle calculation [21, 23]. In the case of NMRooting [21] that has a 3D vectorization function, RSA is vectorized by tracing the shortest path from each root voxel to the root–shoot junction, followed by pruning the short paths that are not on the root segments. However, this vectorization algorithm is sensitive to the noise in 3D volumes. Generally, there is a tradeoff among scanning time, scanning area size, and image quality in image acquisition [24–27]. Therefore, rapid scanning of a large-sized pot tends to make noisy image data. Additionally, minerals, air, and water inside soils also become noise [26]. For example, the root-segmented images in a study conducted on RSAvis3D have much noise [20]. This is because RSAvis3D was conditioned to be applicable to high-throughput RSA visualization, thereby deprioritizing the image quality; it scanned the relatively large pots using the calcined clay [28] (having large voids between particles) for a short time. Moreover, X-ray CT images of undisturbed soil samples collected from the fields [29] also contain non-root segments as noise. In such cases, a vectorization software with a robust algorithm that is not affected by noise is needed.

In this study, based on the root-segmented images using RSAvis3D [20], we developed a robust vectorization software RSAtrace3D for measuring monocot RSA. By indicating some base points that represent where the roots are located, RSAtrace3D vectorizes monocot RSA comprising radicle and crown roots even in noisy images. Further, we vectorize rice (*Oryza sativa*) RSA and measure RSA-related traits, such as root length, root growth angle, and root distribution parameters in the soil. We demonstrate that these measurements are equal to or more accurate than those performed using hand or a 2D vectorization.

### Implementation

**Fig. 1:**
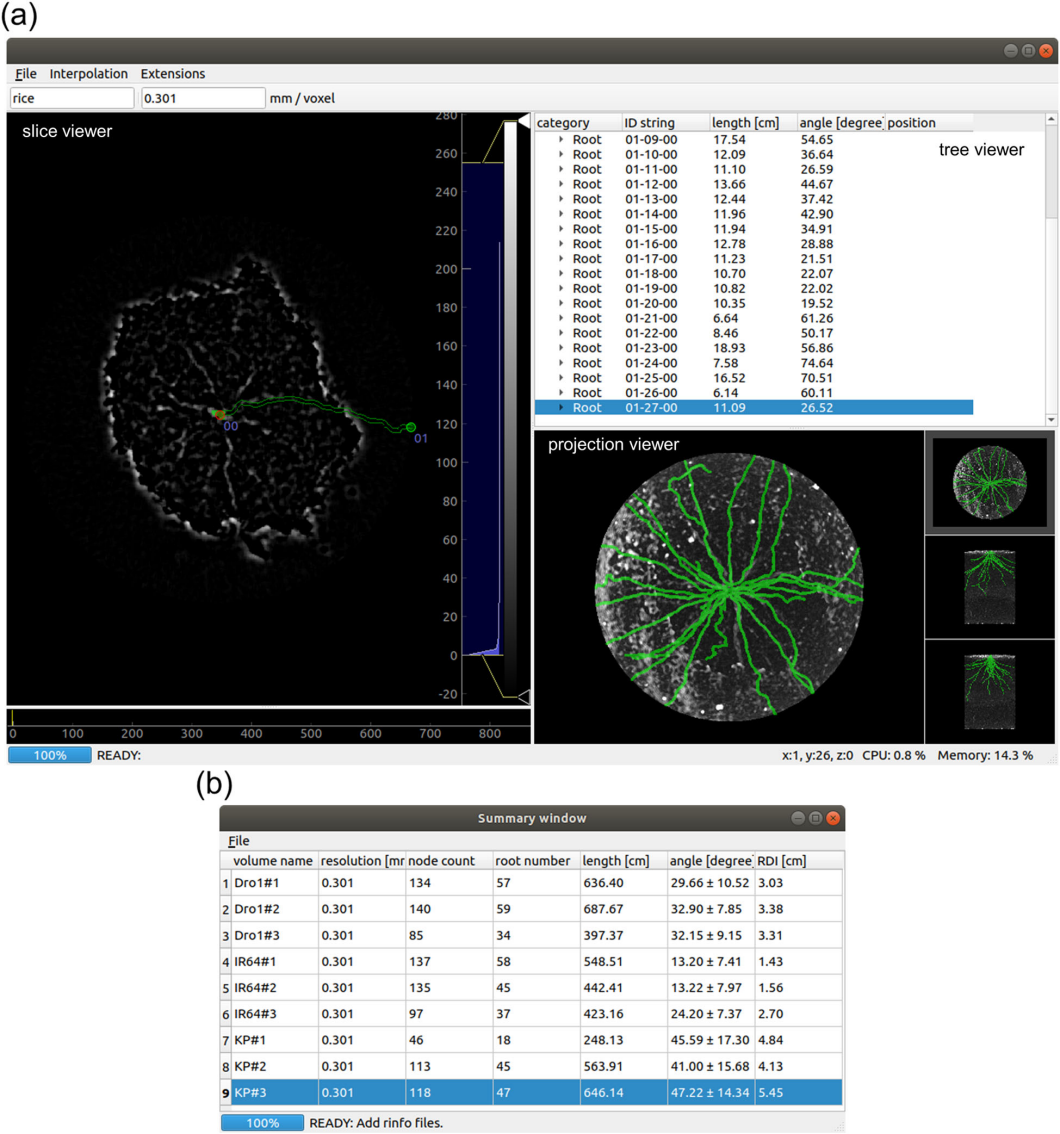
The GUI of RSAtrace3D. (**a**) The main windows comprise the slice, tree, and projection viewers. The slice viewer illustrates a horizontal plane of the volume, of which the position can be freely changed. The tree viewer shows single root-related traits, and its data are exportable. The projection viewer shows a projection image that helps users’ operation. (**b**) The summary window shows RSA-related traits, and its data are exportable

RSAtrace3D is a graphical user interface (GUI) software implemented in Python3 [30] (Fig. 1(a)). It has a main window, including three subviewers (the slice, tree, and projection viewers)—to show the operators processed images and data, and it enables one to operate CT image analysis visually and intuitively. It shows a volume slice image, which is selected in any position by an operator. Further, RSAtrace3D requires base points to make the vectorization in the slice image, and the operator places them as “nodes” in the slice viewer. Thus, RSAtrace3D vectorizes the roots using the given nodes and calculates single root-related traits. The results are shown in the tree viewer. The projection viewer helps us to observe the overall progress of the vectorization. Additionally, the vectorized RSA is saved as a JavaScript Object Notation, (JSON) [31] formatted file as “root information (rinfo).” RSA-related traits calculated from the rinfo files are presented in the summary viewer (Fig. 1(b)).

#### Overview of RSAtrace3D

**Fig. 2:**
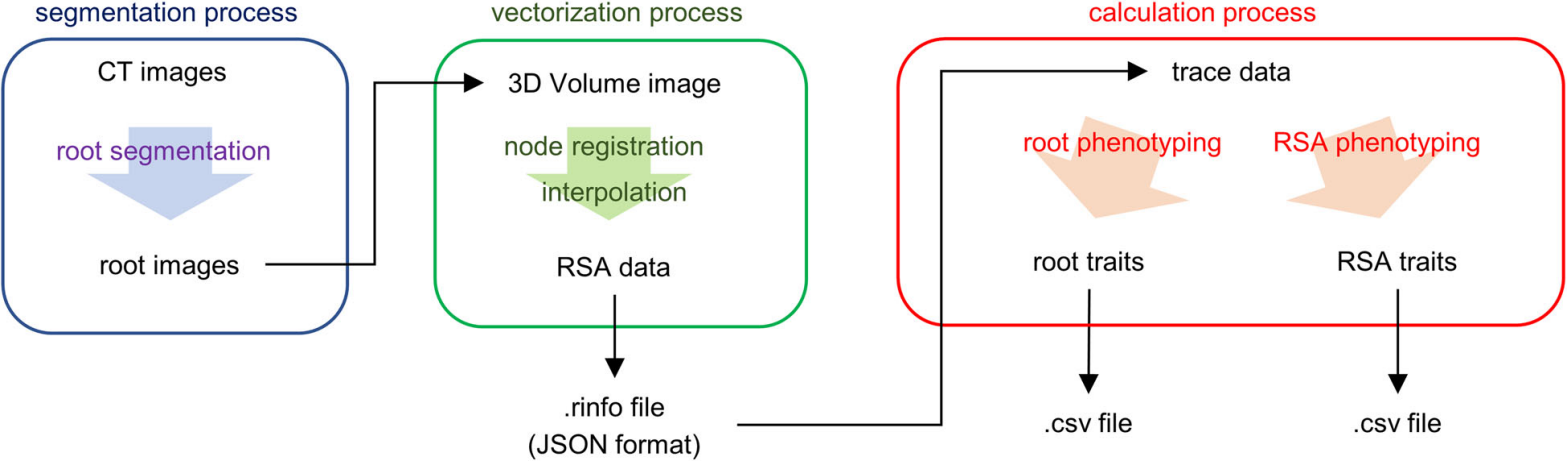
Schematic diagram of the process and data flow in RSAtrace3D. In the segmentation process, CT images are processed using root segmentation software to make root-segmented images. In vectorization process, the root images are imported as a 3D volume image. Besides, operators indicate where the roots are by registering the nodes, which are interpolated and smoothly connected to make a vector. The vectorized data are exportable as rinfo files (JSON format). In the calculation process, root- and RSA-related traits are calculated using the vectorized data

Process and data flow in RSAtrace3D are demonstrated in Fig. 2, which is divided into three processes: segmentation, vectorization, and calculation processes. In the segmentation process, root segments are isolated from raw CT images. This function is not handled by RSAtrace3D and should be performed using root segmentation software, such as RSAvis3D [20]. In the vectorization process, root segments are vectorized by applying GUI operations. Operators register base points (nodes), such as both ends of the root segments, which are vectorized by the interpolation between the nodes. Vectorization is performed on individual roots. In addition, vectorized data are stored as a rinfo file. In the calculation process, single root- and RSA-related traits are calculated using the vectorized data, and they are exportable as comma-separated value (CSV) files.

#### Segmentation process

Volumetric data, which should be 8-bit grayscale image and stored in a single directory, are imported as a 3D NumPy array [32]. It is recommended to volumetric data in which root fragments are enhanced by utilizing root vectorization software, such as RSAvis3D [20].

#### Vectorization process

##### RSA data formatting

**Fig. 3:**
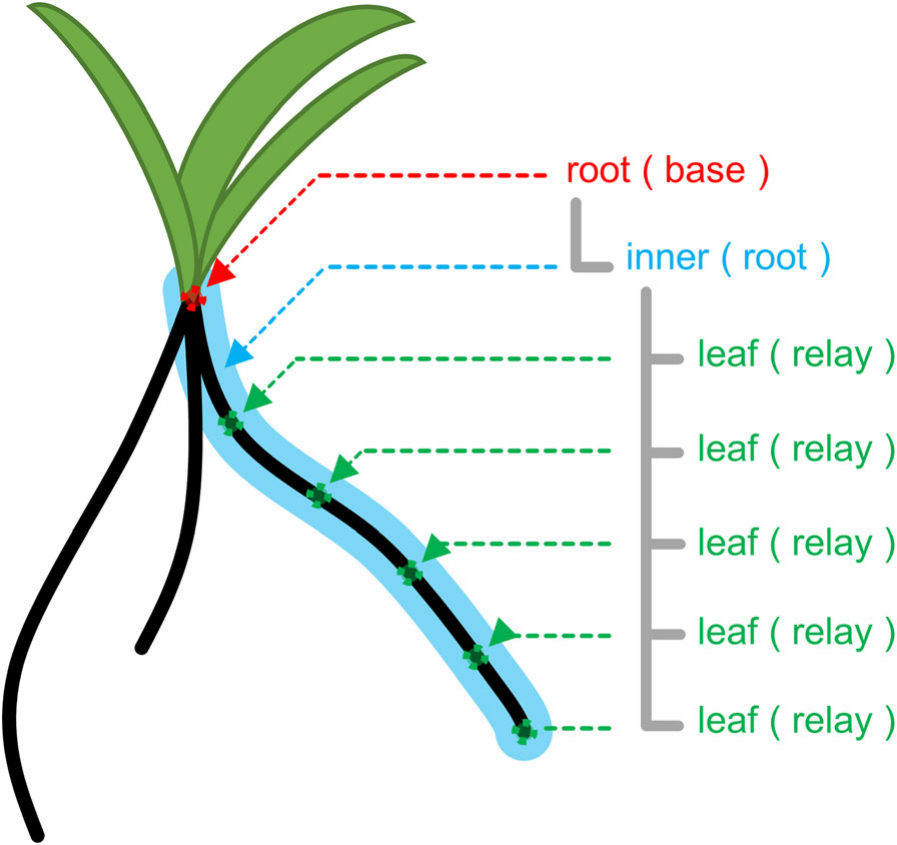
RSA data management in RSAtrace3D. RSA is defined using a tree structure comprising the root, inner, and leaf nodes. The root node represents the place where seeds are sown. The inner node contains the leaf nodes representing the location of root segments. The inner node represents a single root structure

RSA vectorized data are managed using a tree structure object (Fig. 3). In this case, the depth of the tree is 2. Location of roots in the soil is represented in Euclidean coordinates. The root node (depth 0) contains the information of all single roots comprising RSA. The root node contains the coordinate of the place where seeds are sown. The inner node (depth 1) contains the single root-related information, comprising several leaf nodes (depth 2) containing the coordinate that indicates the location of root fragments. For example, an RSA having 1 radicle and 2 crown roots is represented by 1 root node containing 3 inner nodes. In this study, root, inner, and leaf nodes are henceforth written as the base, root, and relay nodes, respectively.

##### Line making and interpolation between nodes

**Fig. 4:**
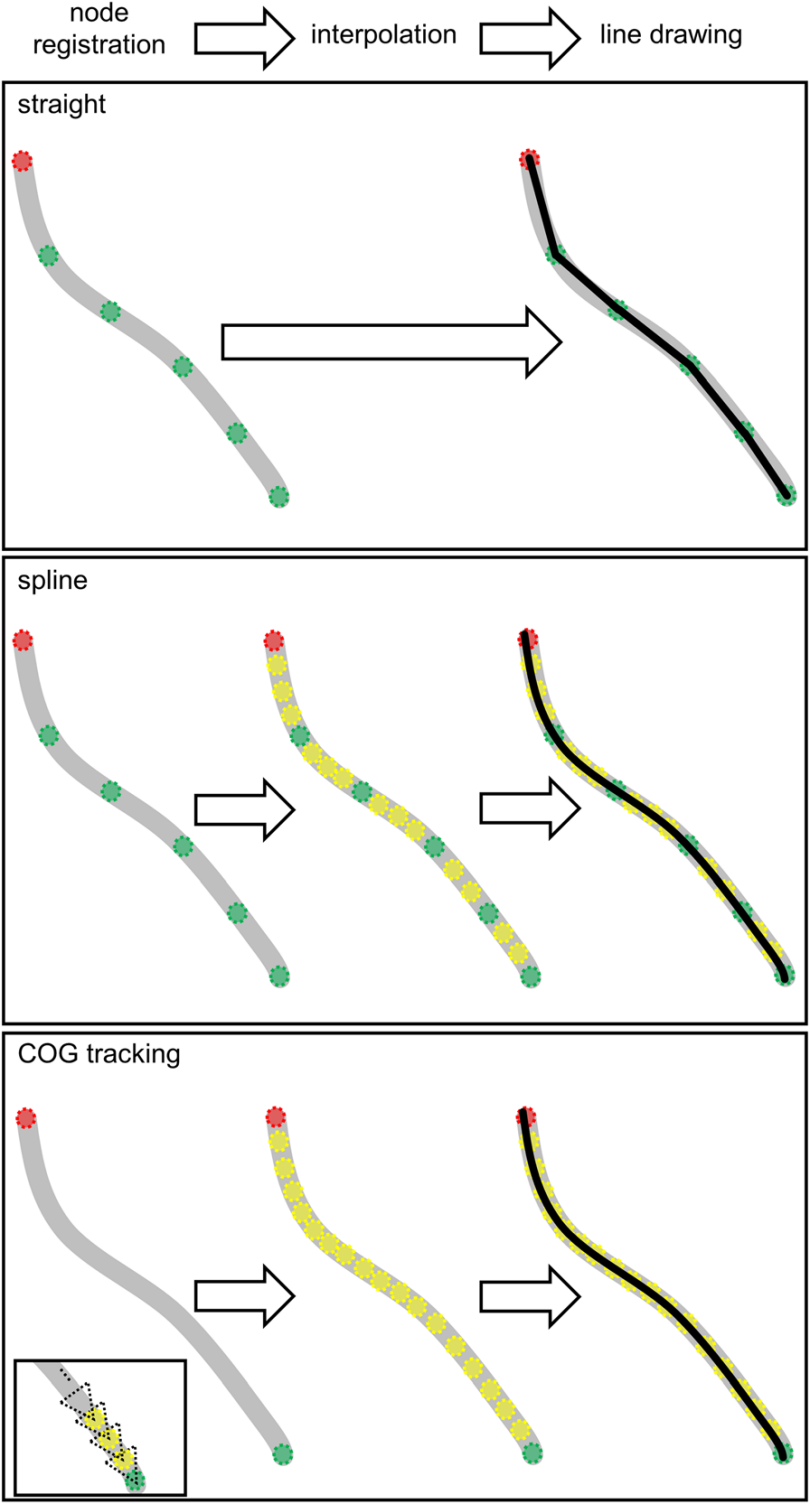
Three vectorization algorithms. “Straight,” “Spline,” and “COG tracking” were implemented. The red, green, and yellow circles indicate the base, registered, and interpolated nodes, respectively. In “COG tracking,” from the terminal node on the other side of the base node, the COG at the narrow area toward the base node (the dashed triangles) is calculated and interpolates the node at the COG-based position, which is repeated until the base node is reached. Polylines are drawn by connecting the adjacent nodes

RSAtrace3D vectorized a single root according to coordinates of the base and relay nodes, making line-connecting nodes to represent the single root shape. Three options are available to make lines: “Straight,” “Spline,” and “COG tracking” (Fig. 4). “Straight” connects the adjacent nodes to make a straight polyline. To represent a complex curve of a root, many relay nodes need to be registered. By automatically adding nodes (interpolated nodes) using a function “splprep” in a module “interpolate” of SciPy, “Spline” makes a spline curve through all base and relay nodes [33]. It gives a smooth line, but a line shape is forced to be a spline curve. “COG tracking” makes a curve by automatically tracking a single root from the node farthest from the base node to the base node through the remaining nodes. Every 4 voxels in the direction of travel is corrected by utilizing the COG of the root segment, and an interpolated node is added. Additionally, “COG tracking” also gives a smooth line and is likely to fit the original root curve. This interpolation process (algorithm) can be customizable and extendable for user’s demands.

#### Calculation processes

##### Single root- and RSA-related trait calculation processes

Single root-related traits: Single root-related traits are measured by employing node and volume information. RSAtrace3D makes the vectorized root data from the nodes. Thus, it is rapid and easy to calculate root shape and size measuring. For example, the root growth angle (the angle between the horizontal line and line connecting both end nodes) and length were calculated in this study. The root growth angle is an angle calculated by applying a trigonometric function, and the root length is the cumulative distance between adjacent nodes. Given that *n* denotes the number of interpolated nodes of single root and Euclidean coordinates of *i*-th node is denoted by *P*_*i*_(*x*_*i*_, *y*_*i*_, *z*_*i*_), the root growth angle *θ* (radian) is calculated by utilizing the following equation:

1 $$\theta ={\mathit{\tan}}^{-1}\left\{\frac{\left|{z}_n-{z}_1\right|}{\sqrt{{\left({x}_n-{x}_1\right)}^2+{\left({y}_n-{y}_1\right)}^2}}\right\}$$

Given that voxel resolution is denoted by *r* (mm/voxel), root length *L* (cm) is calculated using the following equation:

2 $$L=\frac{r}{10}\sum \limits_{i=1}^{n-1}\sqrt{{\left({x}_{i+1}-{x}_i\right)}^2+{\left({y}_{i+1}-{y}_i\right)}^2+{\left({z}_{i-1}-{z}_i\right)}^2}$$

RSA-related traits: Given that *N* denotes the number of single roots and *I*-th root growth angle is denoted by *θ*_*I*_, the average root growth angle $\overline{\theta}$ is calculated by utilizing the following equation:

3 $$\overline{\theta}=\frac{1}{N}\sum \limits_{I=1}^N{\theta}_I$$

Given that *I*-th root length is denoted by *L*_*I*_, the total root length *TL* (cm) is calculated by employing the following equation:

4 $$TL=\sum \limits_{I=1}^N{L}_I$$

Root distribution index (RDI) is a parameter representing how deep a plant proliferates its root [34–36]. It is the centroid of vertical root distribution, and it is calculated by measuring the root length in the soil by depth averaging them weighted with the depth [34, 35]. Before RDI calculation, between nodes is interpolated at a voxel resolution. Given that the number of interpolated nodes in *I*-th root is $\overset{\acute{\mkern6mu}}{n_I}$, vertical Euclidean coordinate of *i*-th node in *I*-th root is denoted by *z*_(*I*, *i*)_, and vertical Euclidean coordinate of the base node is denoted by *z*_*b*_, RDI (cm) is calculated by utilizing the following equation:

5 $$\mathrm{RDI}=\frac{r}{10}\left[\frac{1}{N}\sum \limits_{I=1}^N\left\{\frac{1}{\overset{\acute{\mkern6mu}}{n_I}}\sum \limits_{i=1}^{\overset{\acute{\mkern6mu}}{n_I}}{z}_{\left(I,i\right)}\right\}-{z}_b\right]$$

## Results and discussion

### Root vectorization

**Fig. 5:**
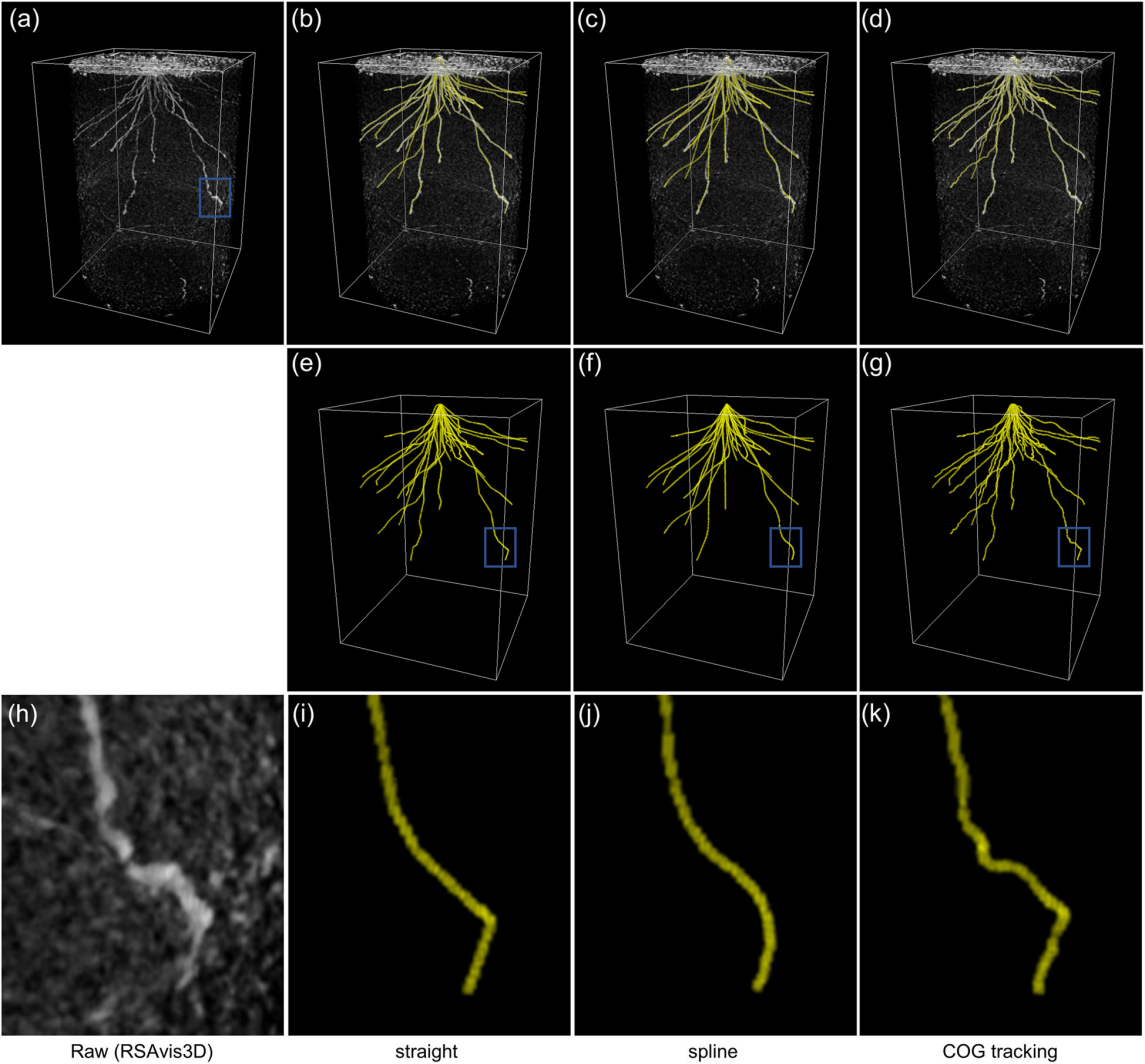
Comparison of tracing performance among the three vectorization methods. The CT images were processed by employing RSAvis3D and vectorized by applying RSAtrace3D. (**a**) 3D rendering of s roots of 26-DAS KP in an X-ray CT image. The X-ray CT image (**a**) was merged with the vectorized polylines by (**b**) “Straight,” (**c**) “Spline,” and (**d**) “COG tracking.” The vectorized polylines are colored in yellow. The vectorized polylines (**e**), (**f**), and (**g**) were isolated from those of (**b**), (**c**), and (**d**), respectively. The enlarged version of the interior, circled by the blue square, in (**a**), (**e**), (**f**), and (**g**) are shown in (**h**), (**i**), (**j**), and (**k**), respectively. The rectangle indicates (width) 180 mm × (depth) 180 mm × (height) 258 mm

The radicle and crown roots of a rice cultivar, Kinandang Patong (KP, ssp. tropical *japonica*), were vectorized using RSAtrace3D. To enhance root segments of 26 days after sowing (26-DAS) KP [20], an X-ray CT image processed using RSAvis3D was used. The 3D rendered CT image of KP, which is colored in white, is shown in Fig. 5(a), and those merged with vectorized polylines, colored in yellow, by “Straight,” “Spline,” and “COG tracking” are shown in Fig. 5(b)–(d). When the drawn line was inside the original root, it was colored in white. Moreover, the overall root distribution was similar among the three methods (Fig. 5(e)–(g)) but differences were observed in the details (Fig. 5(h)–(k)). Compared with the polylines by “Straight” and “Spline” (Figs. 1(i) and (j)), the polyline by “COG tracking” closely traced the original root segment (Fig. 5(h) and (k)). The required click numbers and time for vectorizing by “Straight,” “Spline,” and “COG tracking” are presented in Table 1. The “COG tracking” required 10 min for a sample but required over 50% fewer both for clicks and time than in “Straight” or “Spline” being the least laborious method. In general, “COG tracking” is the most precise and least laborious method compared with “Straight” and “Spline.”

**Table 1:** Comparison of the required labor among the three vectorization methods. The required click number and time to vectorize the root segments of 26-DAS KP in an X-ray CT image

Vectorization method	Required click number	Required time [min]
Straight	284	40
Spline	120	20
COG tracking	56	10

### Measurement application

To evaluate the measurement accuracy, we compared the root growth angle and length between manual measurement and three functions in RSAtrace3D. The following three rice lines were used: IR64 (ssp. *indica*), Dro1-NIL (ssp. *indica*), and KP. Besides, IR64 has shallow roots, whereas KP has deep roots. Dro1-NIL has an intermediate-rooting angle between IR64 and KP. Additionally, it is a near-isogenic line of IR64 introducing the KP allele at *DEEPER ROOTING 1* (*DRO1*) gene, which was isolated from a biparental population between IR64 and KP as a contributor of the deep-rooting property of KP. The wired basket method [20] was utilized for root sampling in the soil substrate. The wired basket, which contained several wires inside and kept the 3D structure of the RSA when removing the soil, was buried in the pot. Four each of IR64, Dro1-NIL, and KP seeds were sown in a calcined clay, which is a soil substrate, inside the wired basket. After 4 weeks, the pot was scanned using an X-ray CT, and the wired basket was excavated from the pot. The X-ray CT images were processed by applying RSAvis3D, and the resulting root images were imported by utilizing RSAtrace3D to vectorize radicle and crown roots. By employing manual measurements, the root growth angle was measured based on where the roots were emanating from the basket mesh. Besides, the roots inside the basket were collected and scanned using a scanner (Expression 12000XL, Seiko Epson Corporation, Suwa, Nagano, Japan), and the root length was measured using SmartRoot [23].

**Fig. 6:**
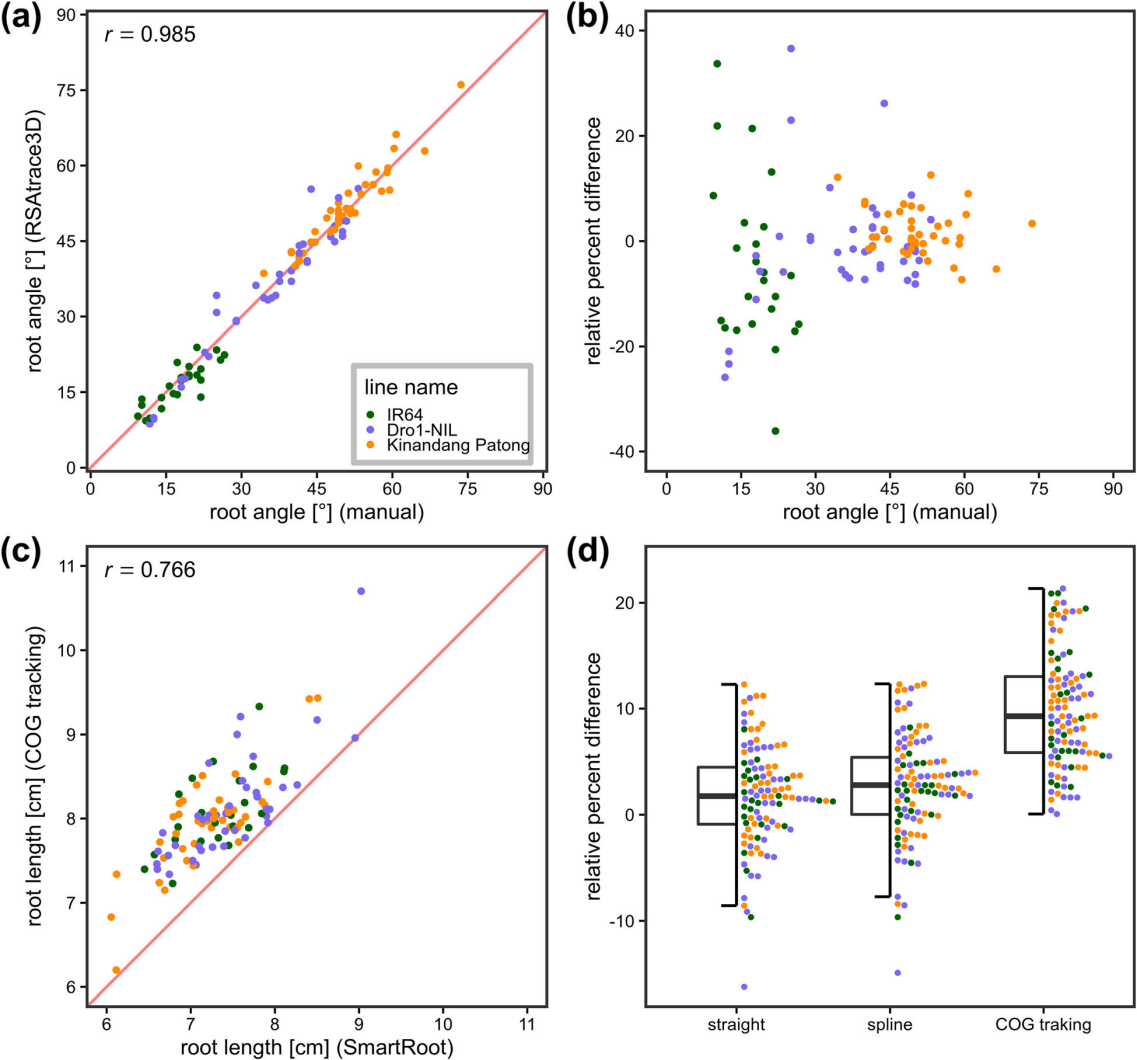
Measurement comparison between manual and RSAtrace3D methods. (**a**) A scatter plot of the root growth angle (an angle between both end nodes) of each root. The identity line is shown in red. Pearson’s correlation coefficient is shown. (**b)** RPDs of the root growth angle (“COG tracking” measurements in RSAtrace3D against manual measurements). (**c**) A scatter plot of root length of each root. The identity line is shown in red. Pearson’s correlation coefficient is shown. (**d**) RPDs of the root length (“COG tracking” measurements in RSAtrace3D against manual measurements). Three methods “Straight,” “Spline,” and “COG tracking” were compared. For the box plots, its top and bottom mark the first and third quartiles, respectively. Further, the center line represents the median, and the whiskers show the range of observed values within 1.5 times the interquartile range from the hinges

The manual and RSAtrace3D measurements were compared (Fig. 6). In the root growth angle, a high positive correlation was observed throughout all three lines (Fig. 6(a), *R* = 0.985). The variance of the relative percent difference (RPD) between the manual and RSAtrace3D measurements became larger in the shallow angular region (Fig. 6(b)). This is because the effect of the unit error increases as the angle decreases. In the case of measuring an average root growth angle from multiple roots, there is little influence of the high variance of RPD since the latter was symmetrical on the x-axis. In root length, a positive correlation was observed throughout all three lines (*R* = 0.766), and, compared with manual measurements, the RSAtrace3D measurements were high in all cases (Fig. 6(c)). We calculated the RPD of RSAtrace3D against SmartRoot in the cases of using “Straight,” “Spline,” and “COG tracking” methods (Fig. 6(d)). The medians of RPD in “Straight” and “Spline” were approximately 0% while that in “COG tracking” was approximately 10%. Consequently, this difference comes from the precise tracking of 3D wavy roots by utilizing RSAtrace3D (Fig. 5(h)–(k)). Moreover, “Straight” and “Spline” would underestimate the root length. Scanning in 2D with 3D wavy roots will similarly underestimate the root length (Fig. 6(c) and (d)). Overall, “COG tracking” in RSAtrace3D is the most reliable way to measure 3D wavy roots.

### A case study: different rooting type of three rice lines

**Fig. 7:**
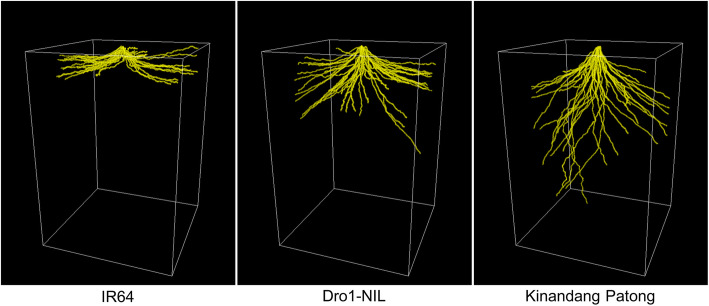
Three-dimensional rendering of IR64, Dro1-NIL, and KP roots. The CT images were processed and vectorized by applying RSAvis3D and RSAtrace3D, respectively. The cube indicates (width) 180 mm × (depth) 180 mm × (height) 258 mm

**Fig. 8:**
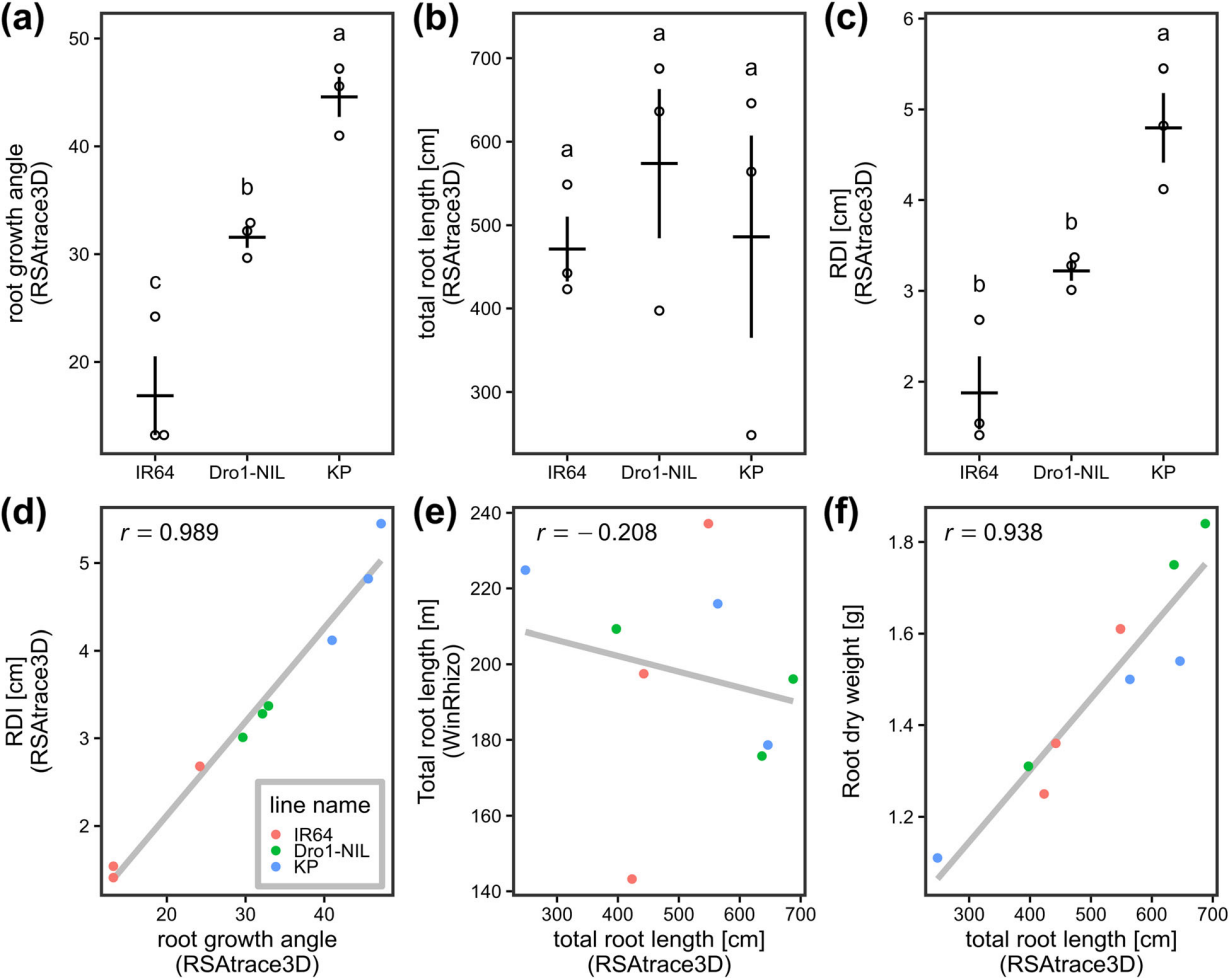
Measurements using RSAtrace3D and its comparison with those of the traditional method. (**a**) Root growth angle (an angle between both end nodes), (**b**) total root length, and (**c**) RDI of IR64, Dro1-NIL, and KP. The horizontal and vertical lines indicate the means and ranges of the standard error, respectively (sample size = 3). Each alphabet indicates a statistically significant difference (Tukey–Kramer’s test, *α* = 0.05). Comparison of (**d**) root growth angle vs. RDI, (**e**) total root length using RSAtrace3D vs. ones using WinRhizo, and (**f**) total root length using RSAtrace3D vs. RDW. The lines indicate the linear regression lines. Pearson’s correlation coefficient is shown

Root parameters of IR64, Dro1-NIL, and KP were quantitively evaluated using RSAtrace3D. Three each of IR64, Dro1-NIL, and KP were cultivated for 2 months and subjected to X-ray CT scanning. The CT images were processed by applying RSAvis3D and vectorized by utilizing RSAtrace3D. After CT scanning, roots in the soil were collected, and their total root length was measured using WinRHIZO™ Pro 2017a software (Regent Instruments, Canada). The scanned roots were dried in an oven, and their root dry weight (RDW) was measured. The representative RSA images are demonstrated in Fig. 7. As previously reported, different root distribution properties were observed. Root growth angle, total root length, and RDI of three rice lines were automatically calculated by employing RSAtrace3D (Fig. 8(a)–(c)). Root growth angles in three lines were different (Fig. 8(a)), whereas root length in three lines was similar (Fig. 8(b)). In addition, RDIs in three lines were different (Fig. 8(c)). There was a high correlation between root growth angle and RDI (Fig. 8(d)), indicating that the RDI depended on the root growth angle. The total root length measured by utilizing RSAtrace3D did not correlate with that of WinRHIZO measurements (Fig. 8(e)). In this study, to exclude the roots touching the wall from RSA shape-related data, we visualize only a cylinder region of 18-cm diameter in the utilized pot with a 20-cm diameter using RSAvis3D. Moreover, the total root length by employing RSAtrace3D was highly correlated with the RDW (Fig. 8(f)). It was considered that the contribution of the rootbound not being visualized using RSAvis3D, of which the majority comprised thin roots to dry weight of total roots is small.

## Conclusions

RSAtrace3D is an RSA vectorization software for 3D RSA phenotyping for monocots. By introducing a COG-based tracking algorithm, we observed that the vectorization process is four times faster than that of simply connecting the nodes using a straight polyline. Depending on the sample size of the root system, the time required for rice RSA vectorization was approximately 10 min. For example, RSAvis3D visualized rice RSA for 15 min [20], including scanning and reconstruction time. In this case, it is possible to vectorize RSA of the previous CT scan while waiting for the scan to complete. When processing a large number of root samples, this high-throughput pipeline of image analysis by employing the RSAvis3D and RSAtrace3D is useful. Once converted to a vector, a very small amount of data is required to represent the root system. In the case study, we utilized three each of three rice lines as subjects. Thus, the number of samples is nine, and its total data size is approximately 16 GB in X-ray CT image (16-bit tagged image file format (TIFF)), 800 Mb in RSAvis3D images (8-bit portable network graphics (PNG)), and 500 Kb in RSAtrace3D vector data. This small data size makes it easy to share data among researchers, thereby facilitating reanalysis and consequently accelerating root research. Owing to the high expandability of the RSA vectorization and phenotyping algorithm, RSAtrace3D could be applied not only to rice in X-ray CT images but also to other monocots in various 3D images. The RSAtrace3D program may be obtained from [37].

## Availability and requirements

Project name: ROOTomics.

Project home page: https://rootomics.dna.affrc.go.jp/en/

Operating system(s): Cross platform.

Programming language: Python3.

Other requirements: Python 3.6 or higher.

License: MIT license.

Any restrictions to use by non-academics: Commercial license needed.

## Data Availability

The plant materials and raw data of the figures used in this study are available from the corresponding author on reasonable request.

## Abbreviations

RSA: Root system architecture
CT: Computed tomography
MRI: Magnetic resonance imaging
COG: Center of gravity
RDI: Root distribution index
KP: Kinandang Patong
DAS: Days after sowing
*DRO1*: *DEEPER ROOTING 1*
RPD: Relative percent difference
RDW: Root dry weight
GB: Gigabyte

## References

[1] Lynch J (1995). Root architecture and plant productivity. Plant Physiol.

[2] Smith S, De Smet I. Root system architecture: insights from Arabidopsis and cereal crops. Philos Trans Royal Soc B: Biol Sci. 2012;367:1441–52. 10.1098/rstb.2011.0234 PMC3321685 22527386

[3] Barley K (1970). The configuration of the root system in relation to nutrient uptake. Adv Agron.

[4] Gardner W (1964). Relation of root distribution to water uptake and availability. Agron J.

[5] de Dorlodot S, Forster B, Pagès L, Price A, Tuberosa R, Draye X (2007). Root system architecture: opportunities and constraints for genetic improvement of crops. Trends Plant Sci.

[6] Gowariker V, Krishnamurthy VN, Gowariker S, Dhanorkar M, Paranjape K (2009). The fertilizer encyclopedia.

[7] Ludlow M, Muchow R (1990). A critical evaluation of traits for improving crop yields in water-limited environments. Adv Agron.

[8] Gowda VR, Henry A, Yamauchi A, Shashidhar H, Serraj R (2011). Root biology and genetic improvement for drought avoidance in rice. Field Crops Res.

[9] Böhm W (1979). Methods of studying root systems.

[10] Zhu J, Ingram PA, Benfey PN, Elich T (2011). From lab to field, new approaches to phenotyping root system architecture. Curr Opin Plant Biol.

[11] Yoshino K, Numajiri Y, Teramoto S, Kawachi N, Tanabata T, Tanaka T, Hayashi T, Kawakatsu T, Uga Y (2019). Towards a deeper integrated multi-omics approach in the root system to develop climate-resilient rice. Mol Breed.

[12] Heeraman DA, Hopmans JW, Clausnitzer V (1997). Three dimensional imaging of plant roots in situ with X-ray computed tomography. Plant Soil.

[13] Schulz H, Postma JA, van Dusschoten D, Scharr H, Behnke S. Plant root system analysis from MRI images. In: Computer Vision, Imaging and Computer Graphics Theory and Application. Springer; 2013. p. 411–25.

[14] Clark RT, MacCurdy RB, Jung JK, Shaff JE, McCouch SR, Aneshansley DJ, Kochian LV (2011). Three-dimensional root phenotyping with a novel imaging and software platform. Plant Physiol.

[15] Metzner R, Eggert A, van Dusschoten D, Pflugfelder D, Gerth S, Schurr U, Uhlmann N, Jahnke S (2015). Direct comparison of MRI and X-ray CT technologies for 3D imaging of root systems in soil: potential and challenges for root trait quantification. Plant Methods.

[16] Mairhofer S, Zappala S, Tracy SR, Sturrock C, Bennett M, Mooney SJ, Pridmore T (2012). RooTrak: automated recovery of three-dimensional plant root architecture in soil from x-ray microcomputed tomography images using visual tracking. Plant Physiol.

[17] Mairhofer S, Zappala S, Tracy S, Sturrock C, Bennett MJ, Mooney SJ, Pridmore TP (2013). Recovering complete plant root system architectures from soil via X-ray μ-computed tomography. Plant Methods.

[18] Tracy SR, Black CR, Roberts JA, McNeill A, Davidson R, Tester M, Samec M, Korošak D, Sturrock C, Mooney SJ (2012). Quantifying the effect of soil compaction on three varieties of wheat (*Triticum aestivum* L.) using X-ray micro computed tomography (CT). Plant Soil.

[19] Gao W, Schlüter S, Blaser SR, Shen J, Vetterlein D (2019). A shape-based method for automatic and rapid segmentation of roots in soil from X-ray computed tomography images: Rootine. Plant Soil.

[20] Teramoto S, Takayasu S, Kitomi Y, Arai-Sanoh Y, Tanabata T, Uga Y (2020). High-throughput three-dimensional visualization of root system architecture of rice using X-ray computed tomography. Plant Methods.

[21] van Dusschoten D, Metzner R, Kochs J, Postma JA, Pflugfelder D, Bühler J, Schurr U, Jahnke S (2016). Quantitative 3D analysis of plant roots growing in soil using magnetic resonance imaging. Plant Physiol.

[22] Gao W, Blaser SR, Schlüter S, Shen J, Vetterlein D (2019). Effect of localised phosphorus application on root growth and soil nutrient dynamics in situ–comparison of maize (*Zea mays*) and faba bean (*Vicia faba*) at the seedling stage. Plant Soil.

[23] Lobet G, Pagès L, Draye X (2011). A novel image-analysis toolbox enabling quantitative analysis of root system architecture. Plant Physiol.

[24] Pierret A, Kirby M, Moran C. Simultaneous X-ray imaging of plant root growth and water uptake in thin-slab systems. In: Roots: The Dynamic Interface Between Plants and the Earth. Springer; 2003: 361–373.

[25] Dhondt S, Vanhaeren H, Van Loo D, Cnudde V, Inzé D (2010). Plant structure visualization by high-resolution X-ray computed tomography. Trends Plant Sci.

[26] Mooney SJ, Pridmore TP, Helliwell J, Bennett MJ (2012). Developing X-ray computed tomography to non-invasively image 3-D root systems architecture in soil. Plant Soil.

[27] Plenge E, Poot DH, Bernsen M, Kotek G, Houston G, Wielopolski P, van der Weerd L, Niessen WJ, Meijering E (2012). Super-resolution methods in MRI: can they improve the trade-off between resolution, signal-to-noise ratio, and acquisition time?. Magn Reson Med.

[28] Adams C, Jacobson A, Bugbee B (2014). Ceramic aggregate sorption and desorption chemistry: implications for use as a component of soilless media. J Plant Nutr.

[29] Kuka K, Illerhaus B, Fritsch G, Joschko M, Rogasik H, Paschen M, Schulz H, Seyfarth M. A new method for the extraction of undisturbed soil samples for X-ray computed tomography. J Nondestr Test. 2013:(8).

[30] Python. https://www.python.org/ Accessed 30 Oct 2020.

[31] Json format. https://www.json.org/json-en.html Accessed 30 Oct 2020.

[32] Walt Svd, Colbert SC, Varoquaux G. The NumPy array: a structure for efficient numerical computation. Comput Sci Eng. 2011;13:22–30.

[33] Jones E, Oliphant T, Peterson P. SciPy: open source scientific tools for Python https://www.scipy.org. Accessed 30 Oct 2020.

[34] Oyanagi A, Nakamoto T, Wada M (1993). Relationship between root growth angle of seedlings and vertical distribution of roots in the field in wheat cultivars. Jpn J Crop Sci.

[35] Oyanagi A (1994). Gravitropic response growth angle and vertical distribution of roots of wheat (*Triticum aestivum* L.. Plant Soil.

[36] Izumi Y, Uchida K, Iijima M (2004). Crop production in successive wheat-soybean rotation with no-tillage practice in relation to the root system development. Plant Prod Sci.

[37] ROOTomics. https://rootomics.dna.affrc.go.jp/en/ Accessed 30 Oct 2020.

